# Estradiol Detection for Aquaculture Exploiting Plasmonic Spoon-Shaped Biosensors

**DOI:** 10.3390/bios13040432

**Published:** 2023-03-29

**Authors:** Francesco Arcadio, Mimimorena Seggio, Luigi Zeni, Alessandra Maria Bossi, Nunzio Cennamo

**Affiliations:** 1 Department of Engineering, University of Campania Luigi Vanvitelli, Via Roma 29, 81031 Aversa, Italy; 2 Department of Biotechnology, University of Verona, Strada Le Grazie 15, 37134 Verona, Italy

**Keywords:** spoon-shaped waveguide, estrogen receptor, surface plasmon resonance, estradiol, aquaculture, plastic optical fiber, biosensor

## Abstract

In this work, a surface plasmon resonance (SPR) biosensor based on a spoon-shaped waveguide combined with an estrogen receptor (ERα) was developed and characterized for the detection and the quantification of estradiol in real water samples. The fabrication process for realizing the SPR platform required a single step consisting of metal deposition on the surface of a polystyrene spoon-shaped waveguide featuring a built-in measuring cell. The biosensor was achieved by functionalizing the bowl sensitive surface with a specific estrogen receptor (ERα) that was able to bind the estradiol. In a first phase, the biosensor tests were performed in a phosphate buffer solution obtaining a limit of detection (LOD) equal to 0.1 pM. Then, in order to evaluate the biosensor’s response in different real matrices related to aquaculture, its performances were examined in seawater and freshwater. The experimental results support the possibility of using the ERα-based biosensor for the screening of estradiol in both matrices.

## 1. Introduction

In recent years, increasing attention has been paid to the role of estrogens accumulated in the environment and in the human food chain.

Estrogens are essential hormones for human biology and physiology. They have a role in regulating reproduction, cardiovascular and gastrointestinal functions, bone strength, cognitive behavior, and successful pregnancy systems [1]. However, an excessive estrogen exposure can induce cardiovascular diseases, can increase cancer risk, and are also reported to be involved in disorders of the male reproductive system [2,3].

Estrogens are widely used in aquaculture for the sex reversal of fishes. This practice induces a monosex population and it is applied when the growth rate and/or gain weight are different between the male and female specimens. The monosex population is obtained by a hormonal treatment during the sex differentiation of fishes with a protocol that consists in the use of a variety of estrogens, as reported in [4]. Among the many estrogens that can be used for this purpose, the author emphasizes the convenience of using estradiol rather than synthetic estrogens [4].

Nevertheless, estrogen in fish farming might have harmful consequences, such as posing potential risks to human and environmental health related to hormone-dependent parameters [5]. Indeed, hormones from the aquaculture could be released into the environment due to the incorrect or illegal spillage of water containing residues of medicated feed that remain unconsumed by fish.

The serious effects on human and animal health make it crucial to ensure that estrogen release in the environment from different sources should be at levels below the accepted No-Observed Effect Levels (NOELs) [6,7].

The use of hormones in food-producing animals received different legal regulations in different countries. The European Union prohibits the use of substances with hormonal actions in food-producing-animals, including aquaculture (96/22/EC, 2003/74/EC, 2008/97/EC). Other countries such as the United States, Canada, Australia, New Zealand, and Argentina, allow for the use of natural steroid hormones, such as testosterone, progesterone, and estradiol.

To determine the estradiol residues in environmental and food samples, analytical methods have been developed to quantify this substance in different matrices. In this regard, for the quantitative determination, liquid chromatography (LC) is the most employed technique coupled with several types of detectors. The most common detectors combined with LC are electrochemical, ultraviolet diode array (DAD) [8], fluorescence, UV-Vis, mass spectrometry (MS), and tandem mass spectrometry (MS/MS) [9]. These techniques can reach a limit of detection (LOD) on the order of ng/L [10]. However, all the reported analytical techniques need a complex sample pre-treatment or extraction steps before the analysis that could include procedures that are rather complicated and time-consuming [11].

Recently, the surface plasmon resonance (SPR) phenomenon has gathered great attention for developing methods for biomolecules determination. This optical technique allows for the determination of the refractive index changes occurring at the interface between an ultra-thin metal layer and a dielectric medium [12,13]. Nowadays, SPR is widely coupled with optical fiber sensors and is applied in several analytical techniques to monitor environmental contaminants [14,15,16], and for diagnostic purposes [17,18,19].

SPR optical fiber sensors provide several benefits, including small-sizes, remote sensing capabilities, low costs, and high performance [20,21,22]. Moreover, plasmonic optical fiber probes are combined with different receptors to implement specific sensors useful in several application fields [23,24,25].

The evaluation of estradiol by SPR was introduced by Cheskis et al. [26], exploiting the affinity between the estrogen receptor (ERα) and the consensus DNA sequence (ERE), with or without estrogenic compounds. This interaction can find environmental applications in the direct quantification of estrogens by SPR techniques as reported in [10].

In this work, an ERα-based plasmonic biosensor was produced and characterized, without the use of the ERE DNA sequences. Indeed, the ERα immobilization was realized through an established coupling technique to functionalize a suitable SPR sensor directly with the receptor in order to detect the estradiol. For this purpose, an SPR probe based on a spoon-shaped waveguide, previously designed a characterized [27], was chosen. In fact, thanks to its peculiar shape, the measuring cell, integrated in the platform, appears suitable for on-site measurements without the need of microfluidic systems. Moreover, as demonstrated in [27], the peculiar geometry of the spoon-shaped waveguide can be used to obtain a flexible plasmonic probe, with different sensitivities associated with distinct spoon areas (neck or bowl). In fact, the plasmonic phenomena can be tuned by changing both the sensing region and the way the light is launched/collected to/from the sensor (parallel interrogation or orthogonal interrogation modes) [27].

The proposed biosensor was tested for the determination of an estradiol concentration in buffer and real water samples (seawater and freshwater) in order to evaluate the different responses of the biosensor to different matrices. Moreover, a selectivity test was performed with dexamethasone and progesterone, two structural analogues of estradiol.

## 2. Materials and Methods

### 2.1. Chemicals

The compounds α-lipoic acid, N-(3-dimethylaminopropyl)-N′-ethylcarbodiimide hydrochloride (EDC), N-hydroxysuccinimide (NHS), ethanolamine, 2-(N-morpholino)ethanesulfonic acid (MES), saline phosphate buffer 10 mM with pH 7.4 (PBS), estradiol, Glycine, and sodium chloride were purchased from Merck-Sigma (Darmstadt, Germany). Recombinant Human Estrogen Receptor alpha protein (ERα) (ab82606 abcam) was purchased from Abcam (Cambridge, UK).

### 2.2. SPR Sensor Platform Fabrication

**Figure 1:**
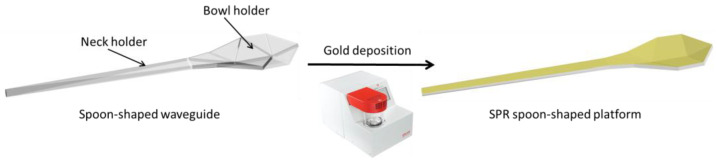
Outline of fabrication process for the SPR sensor based on the spoon-shaped waveguide.

The SPR polymer-based spoon-shaped waveguide fabrication is extensively described in [27]. In summary, the spoon-shaped polymer optical waveguide, schematically represented in Figure 1, had a planar region, on the neck, and a concave bowl, characterized by eight faces [27]. The spoon-shaped waveguide was realized in extruded polystyrene (refractive index (RI) = 1.59 @ 600 nm) supplied by Italia Soft s.r.l. (Trani, Italy).

To produce the SPR sensor based on the spoon-shaped waveguides, only a gold deposition step was necessary. The gold film was deposited by a sputter coater machine (Safematic CCU-010, Zizers, Switzerland) with a final thickness equal to about 60 nm, via three consecutive steps (20 nm/step) to avoid a high increase in the temperature in the sputter chamber. Each deposition step lasted 23 s, with a current of 60 mA and at a working pressure equal to 0.01 mbar. More details about the used plasmonic transducer in terms of the plasmonic phenomena are reported in [27].

### 2.3. ERα Functionalization Protocol

The functionalization of the plasmonic probe was performed in the bowl region of the SPR spoon-shaped platform according to the protocol reported in [28]. This sensing area was selected for its characteristics in terms of its high optical sensitivity and for its peculiar shape acting as a built-in measuring cell [27].

At first, the surface was washed with Milli-Q water (3 times). Then, the gold nanofilm was treated overnight at room temperature with α-lipoic acid (0.3 mM in 8% ethanolic solution) in order to form a self-assembled monolayer (SAM). After an extensive rinsing with Milli-Q water, the carboxylic groups were activated with EDC/NHS (10 mM/10 mM respectively) in an MES buffer (50 mM, pH 5.5) for 20 min at room temperature, according to the immobilization protocol optimized in [28]. The excess of reactant was removed by washing it three times with Milli-Q water. Then, the surface was incubated for 2 h with 40 ng of ERα (16 μL of 2.5 μg/mL stock solution) to immobilize the receptor. Finally, the passivation of the surface was performed by incubating ethanolamine (1 mM in water) for 30 min at room temperature, in order to quench the unreacted activated carboxylic groups. The prepared platforms were washed in PBS and stored submerged in PBS at 4 °C. Each functionalization step was monitored by the changes in the plasmonic spectra calculated with respect to the bare platform (without receptor) considering PBS as a surrounding medium.

### 2.4. Experimental Setup

The presented SPR spoon-shaped probe can be interrogated in different ways to excite multiple plasmonic phenomena, as extensively reported in [27].

**Figure 2:**
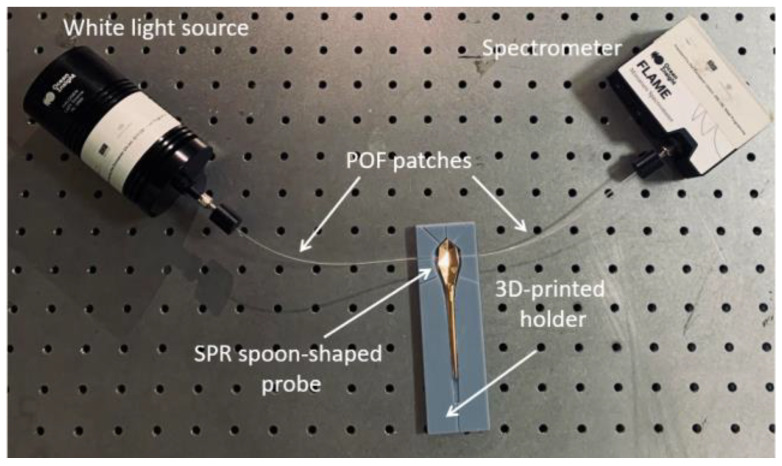
Experimental setup used to test the SPR spoon-shaped platform in orthogonal interrogation mode [27].

In this work, the analysis setup for the specific waveguide was assembled to test the bowl sensitive area of the spoon-shaped probe in the orthogonal interrogation mode [27]. The chosen plasmonic area combined with the orthogonal interrogation mode presents the best optical sensitivity (bulk sensitivity) [27]. Moreover, this plasmonic region contains a built-in measuring cell. As shown in Figure 2, the setup consisted of a halogen lamp as the white light source (HL-2000LL, Ocean Insight, Orlando, FL, USA), a spectrometer (FLAME-S-VIS-NIR-ES, Ocean Insight, Orlando, FL, USA), and the SPR spoon-shaped waveguide platform. Two plastic optical fibers (POFs) patches (∅ = 1 mm) were used to launch and collect the transmitted light, respectively. For the final assembly, the SPR spoon-shaped waveguide sensor was connected to the white light source and the spectrometer by plastic optical fibers (POFs) via a custom 3D-printed holder, produced by a 3D-printer (Photon Mono X UV Resin SLA 3D Printer, Anycubic^®^, Shenzhen, China). The latter was exploited both to connect the SPR spoon-shaped probe with the POFs and to keep it in position during the experimental measurements.

### 2.5. Measurements Procedure

The dose–response curves were obtained in three different matrices: PBS, freshwater, and simulated seawater. Moreover, filtered real freshwater and seawater samples with the addition of estradiol were tested after a 1:20 dilution with PBS. More specifically, a 1 mg/mL stock solution of estradiol was prepared in pure ethanol. Prior the measurements, the stock solution was diluted to the final tested concentrations (ranging from 360 fM to 36 nM) with PBS, with filtered freshwater and with simulated seawater (0.46 M of NaCl in MilliQ water) both diluted 1:20 with PBS.

The real freshwater and seawater samples were collected from an area in Naples’s, far from any kind of aquaculture and/or implant for urban wastewater treatment. The freshwater and seawater samples were consecutively filtered with three different syringe filters (0.8 μm, 0.4 μm, and 0.02 μm) in order to retain the sediment, sand, organic matter, and nanoparticles. Then, a theoretical amount of estradiol (20 pM) was added and finally the samples were diluted with PBS 1:20 before testing.

The samples were also confirmed by a standard method of quantification (JRC/TR EUR 27813:2017).

Measurements on the SPR spoon-shaped sensing platform were performed by placing 150 μL of the water sample (seawater or freshwater) on the bowl-sensing surface and with an incubation time of 4 min. The incubation times were defined according to the preliminary binding kinetics information (more details are reported in the Results and Discussion Section). The plasmonic spectra were collected after a washing step (PBS, twice 150 μL) and by placing PBS (150 μL) as a bulk solution [27].

The experimental data (dose–response curves) were fitted with the Langmuir model equation, which has the general formula herein reported.

(1) $$\left|\Delta \lambda \right|=\left|\lambda_{c}-\lambda_{0}\right|=\left|\Delta \lambda_{max}\right|\cdot \left(\frac{c}{K+c}\right)$$

where *λ_c_* is the resonance wavelength at the analyte concentration *c*; *λ*_0_ is the resonance wavelength in absence of the analyte (blank); Δ*λ_max_* is the maximum value of Δ*λ*, calculated by subtracting the blank value from the saturation value; and K is the ligand concentration at half saturation.

After each measurement, the functionalized platform was regenerated with Glycine/HCl (10 mM, pH 2.2) and stored submerged in PBS at 4 °C.

## 3. Results and Discussion

**Figure 3:**
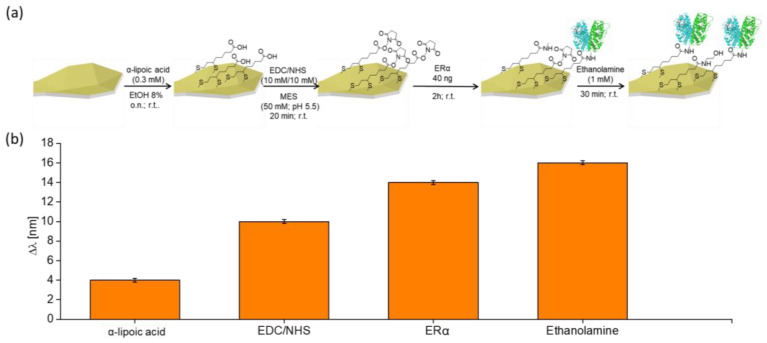
ERα functionalization: (**a**) scheme of functionalization procedure on SPR spoon-shaped probe; (**b**) variation in the resonance wavelength, calculated with respect to the bare chip considering PBS as surrounding medium, after each step of the functionalization process (n = 3, standard deviation (SD) = 0.15 nm).

In order to evaluate the estradiol concentration in the water samples, a sensor based on an SPR spoon-shaped probe was functionalized with ERα, an intracellular receptor able to bind the estradiol with a high affinity constant on the order of pM [29]. The SPR spoon-shaped bowl surface was functionalized with ERα, according to the protocol optimized in [28] with a multi-step procedure as schematically shown in Figure 3a: α-lipoic acid was used to form a SAM; next, the carboxylic acid moieties were activated with EDC/NHS; ERα was covalently coupled to the SAM; finally, the surface was passivated. The successful completion of each functionalization step was monitored by the changes in the plasmonic spectra calculated with respect to the bare platform (without receptor) considering PBS as a surrounding medium. The SPR spectra were obtained by normalizing the spectrum in PBS to the one acquired in air (reference spectrum), where the resonance condition is not satisfied. As shown in Figure 3b, a resonance wavelength variation toward higher values (red shift) could be noticed, in the presence of the same bulk RI (i.e., PBS) after each functionalization step, confirming the effectiveness of the immobilization process. In other words, during the functionalization steps, the refractive index in contact with the gold surface increased and the resonance wavelength shifted on the right.

**Figure 4:**
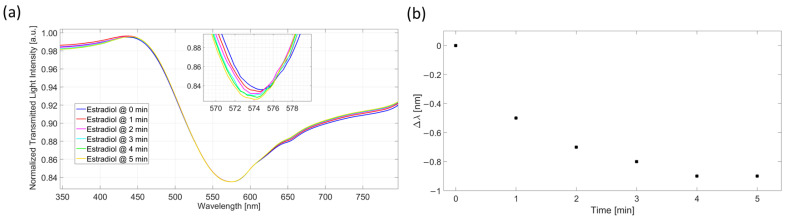
(**a**) Normalized SPR spectra obtained for estradiol (3.6 pM) at different incubation times on ER–SPR spoon-shaped probe. (**b**) Variation in the resonance wavelength (Δ*λ*) as a function of incubation times with respect to initial time (t = 0).

In order to optimize the incubation time, the binding kinetics of the estradiol to immobilized ERα was carried out on the plasmonic platform. As reported in Figure 4, the optimal incubation time was reached in 4 min, after which no changes in the resonance wavelength occurred.

In order to characterize the ER–SPR spoon-shaped biosensor performance, the estradiol binding response of the sensor was tested in PBS at different concentrations ranging from 360 fM to 36 nM.

**Figure 5:**
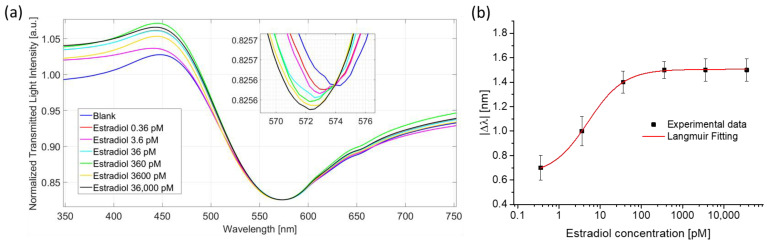
(**a**) Normalized SPR spectra relative to estradiol detection at different concentrations in PBS. (**b**) Absolute value of resonance wavelength variation (with respect to the blank) as a function of estradiol concentration on functionalized platform and Langmuir fitting (red line) of the experimental data on a semi-log scale. The error bars were calculated as standard deviation of the dataset (n = 3).

Figure 5a reports the normalized SPR spectra obtained for different estradiol concentrations at 4 min of incubation. The resonance wavelength decreased for increasing estradiol concentrations. The used spoon-shaped waveguide excited an SPR phenomenon that produced a resonance variation toward the right when the refractive index in contact with the gold nanofilm increased, as shown in the SPR spectra relative to the functionalization process (Figure 3). Nevertheless, the measured SPR shift relative to the receptor–analyte binding suggested that the refractive index of the receptor layer in contact with the gold surface decreased after the binding of the selected analyte.

Indeed, the interaction between the ER and estradiol involved a change in the conformation of the receptor [30] that produced a decrease in the refractive index and, therefore, a blue-shift. This trend is similar to that obtained in [31,32]. Figure 5b reports the binding isotherm along with the fitting of the experimental data with the Langmuir model equation (Equation (1)). The fitting parameters are listed in Table 1.

Moreover, the biosensor was also tested in pure freshwater and simulated seawater in the same estradiol concentration range in order to evaluate the matrix effect on the biosensor response.

**Figure 6:**
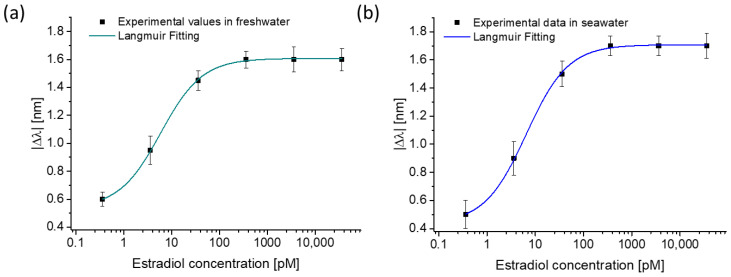
Absolute value of resonance wavelength variation (with respect to the blank) as a function of estradiol concentration on functionalized platform, and Langmuir fitting of the experimental data on a semi-log scale in: (**a**) freshwater, and (**b**) simulated seawater. The error bars were calculated as the standard deviation of the dataset (n = 3).

Furthermore, in these cases, the increase in the estradiol concentrations also produced a decrease in the resonance wavelength. The binding isotherms relative to these matrices are shown in Figures 6a and 6b, respectively. The obtained binding parameters were comparable with the parameter obtained in PBS (see Table 1), supporting the possibility of using an ER-based biosensor for the screening of estradiol also in freshwater and seawater.

The calculated variables reported in Table 1 were used to evaluate the biosensor’s analytical parameters, reported in Table 2, that characterized the proposed estradiol sensor: the sensitivity at a low concentration, limit of detection (LOD), and affinity constant (K_aff_).

It can be inferred from Table 3 that the proposed plasmonic biosensor performances improved with respect to other estradiol SPR-based sensors presented in the literature and combined with different kinds of receptors.

Additionally, the SPR spoon-shaped biosensor was used in the quantification of estradiol in real water samples using the standard additions method.

At first, the collected samples were tested with a standard method of quantification (JRC/TR EUR 27813:2017) performed by an accredited laboratory (Servizi Controllo Qualità s.r.l., Casagiove, Italy) in order to confirm the absence of estradiol in the selected real matrix. The estradiol concentration, originally present in the real matrix sample, was below the limit of detection using the employed technique (LOD < 0.1 ng/L), so that it could be considered as blank. The selected water samples were added with estradiol via spiking with a theoretical amount of estrogen (estradiol 20 pM) and were then diluted to 1:20 with PBS. These prepared samples were then tested with the standard method of quantification mentioned above (JRC/TR EUR 27813:2017), obtaining a final concentration of 1 pM confirming the spiked concentration of estradiol (sample recovery of 100%).

**Figure 7:**
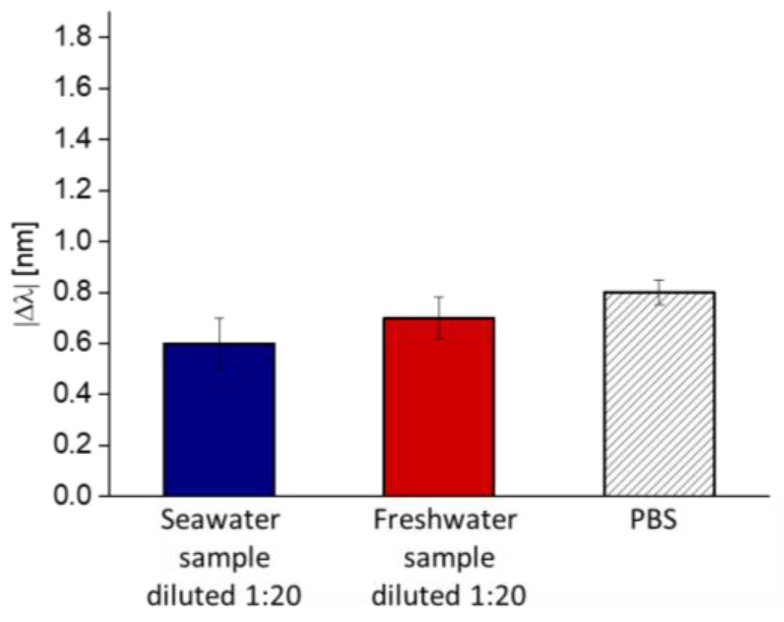
The absolute value of resonance wavelength variation (with respect to the blank) for different matrices with the theoretical addition of estradiol (20 pM). The samples were diluted 1:20 before testing. The error bars were calculated as the standard deviation of the dataset (n = 3).

The concentration of estradiol in such prepared real matrices samples (average of three determinations for each sample) and the recovery of estradiol by the standard addition were measured using the proposed biosensor. The results were summarized in Figure 7. For both the freshwater and seawater samples, the measured wavelength variations were used to evaluate the estradiol concentrations with the calibration curves (reported in Figures 6a and 6b, respectively) obtained for the different matrices. The recovery of all measured samples was 97% for the seawater sample, 113% for the freshwater sample, and 100% in PBS. From the comparison between the PBS and real matrices’ recoveries, it was possible to evaluate this kind of biosensor showing a slight matrix effect. These results indicated that the proposed biosensor system could be applied to estradiol analysis in real environmental water samples.

**Figure 8:**
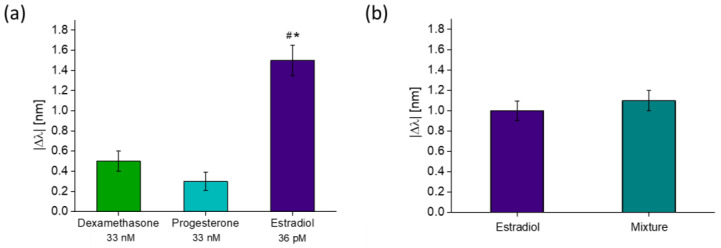
Selectivity test: (**a**) the absolute value of resonance variation for the structural analogues of estradiol (dexamethasone 33 nM and progesterone 33 nM) together with estradiol (36 pM). One-way ANOVA * *p* < 0.05 vs. dexamethasone and # *p* < 0.05 vs. progesterone. (**b**) Comparison between the absolute value of resonance variation for only estradiol 3.6 pM with a mixture of estradiol and the two structural analogues each considered at a concentration of 3.6 pM. The error bars were calculated as a standard deviation of the dataset (n = 3).

Moreover, the selectivity of the ERα SPR spoon-shaped biosensor was examined, evaluating the response in the presence of interfering substances having a molecular structure close to estradiol. For this purpose, dexamethasone and progesterone were chosen as structural analogues to estradiol. The selectivity tests were carried out by testing the analogues in two different ways, i.e., one by one (Figure 8a) and in mixtures (Figure 8b). As shown in Figure 8a, this was possible to evaluate, as dexamethasone and progesterone (individually tested) produced a slight shift in the plasmonic wavelength at a concentration three orders of magnitude greater than the estradiol concentration. The high concentration value of the analogues (33 nM for analogues vs. 36 pM for estradiol) was chosen to obtain measurable resonance shifts.

In the second selectivity test, a mixture of the three compounds was prepared at the same concentration each (3.6 pM). Figure 8b shows that the shift produced by the substances’ mixture (estradiol, dexamethasone, and progesterone at the same concentration) was very similar (within the error bar) to the one produced by the single estradiol considered at the same concentration (3.6 pM).

At last, to assess the repeatability and reproducibility of the proposed biosensor, several tests were performed, in homogeneous conditions, on the same platform and on three different ERα-functionalized platforms, respectively, after cycles of binding tests and regeneration steps.

**Figure 9:**
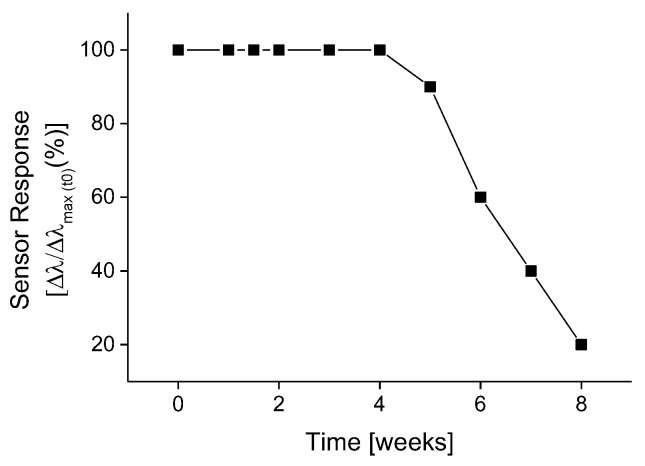
Sensor response (%) during the time. Resonance wavelength variation (Δ*λ*) normalized to the maximal response obtained at the first use (Δ*λ*_max(t0)_) as a function of time during the binding and regeneration step.

After each measurement, the ERα–SPR spoon-shaped biosensor was regenerated in order to release the analyte from the ERα at the surface of the chip and reuse the sensor. The regeneration was realized by an extensive washing step with 10 mM glycine/HCl (10 mM, pH 2.2) while the storage was at 4 °C in PBS. Figure 9 shows the efficiency of the regeneration cycles over time for the same biosensor platform. As reported in Figure 9, the regeneration process of the ERα-functionalized platform was fully successful after five regeneration cycles over one month. These results underline the good reusability of the proposed sensor within this time window. However, then a progressive worsening of the response was observed.

## 4. Conclusions

In this work, the development and characterization of an ERα–SPR spoon-shaped biosensor for the detection and quantification of estradiol in real water samples was presented. The biosensor was obtained by functionalizing the bowl region of an SPR spoon-shaped platform, featuring a built-in measuring cell, with a specific estrogen receptor that was (ERα) able to bind the estradiol. The proposed biosensor was successfully tested in two different real matrices related to aquaculture, i.e., seawater and freshwater, by exploiting an extrinsic POF biosensor. More specifically, the spoon-shaped biochip was monitored to detect estradiol in real samples via POFs connected with a white light source and a spectrometer. Overall, the ERα–SPR spoon-shaped biosensor appeared to be able to detect the estradiol in the range of concentration requested by a real scenario. In the future, the biosensor proposed here may also find applications, other than in environmental monitoring, in hormone quantification in biomedical and pharmaceutical fields.

## Figures and Tables

**Table 1:** Langmuir parameters of estradiol detection in PBS, freshwater, and in simulated seawater.

ER–SPR Spoon-Shaped	∆*λ*_0_ [nm]	∆*λ*_max_ [nm]	K [pM]	Statistics	
				Reduced χ^2^	Adj. R^2^
In PBS	0.64 ± 0.01	1.51 ± 0.01	5.05 ± 0.24	0.01	0.999
In freshwater	0.55 ± 0.01	1.62 ± 0.01	5.89 ± 0.33	0.02	0.999
In simulated seawater	0.43 ± 0.02	1.70 ± 0.01	6.50 ± 0.50	0.03	0.999

**Table 2:** ER–SPR spoon-shaped analytical parameters relative to estradiol detection.

ER–SPR Spoon-Shaped	Sensitivity at Low Concentration (|Δ*λ*_max_|/K)	LOD * (3 × Standard Deviation of Blank (Δ*λ*_0_)/Sensitivity at Low Concentration)	K_aff_ (1/K)
In PBS	0.299 nm/pM	0.10 pM	0.198 pM^−1^
In freshwater	0.275 nm/pM	0.11 pM	0.170 pM^−1^
In simulated seawater	0.261 nm/pM	0.23 pM	0.154 pM^−1^

* from [33].

**Table 3:** Plasmonic sensors for estradiol detection combined with different receptor types.

Transducer	Receptor	LOD [pM]	Reference
highly tilted fiber Bragg grating	hERα ligand-binding domain	5.4	[34]
commercial SPR chip	ERα combined with conformation-sensitive peptides	1000	[35]
commercial SPR chip	11-MUA/E2-BSA conjugate vs. Ab-E2	3.6	[36]
commercial SPR chip	Molecularly Imprinted Polymer (MIP)	0.25	[37]
commercial SPR chip	Antibody combined with magnetic nanoparticles	292	[38]
spoon-shaped waveguides	ERα	0.1	This work

## Data Availability

The data are available on reasonable request from the corresponding author.
